# MrTPS3 and MrTPS20 Are Responsible for β-Caryophyllene and α-Pinene Production, Respectively, in Red Bayberry (*Morella rubra*)

**DOI:** 10.3389/fpls.2021.798086

**Published:** 2022-01-07

**Authors:** Yan Wang, Qinsong Yang, Yifan Zhu, Lan Zhao, Pengju Ju, Guoyun Wang, Chaochao Zhou, Changqing Zhu, Huijuan Jia, Yun Jiao, Huimin Jia, Zhongshan Gao

**Affiliations:** ^1^College of Agriculture and Biotechnology, Zhejiang University, Hangzhou, China; ^2^Key Laboratory for Silviculture and Conservation, Ministry of Education, Beijing Forestry University, Beijing, China; ^3^Yuyao Agriculture Technology Extension Center, Ningbo, China; ^4^Institute of Forestry, Ningbo Academy of Agricultural Science, Ningbo, China; ^5^College of Agronomy, Jiangxi Agricultural University, Nanchang, China

**Keywords:** TPS, VOC, β-caryophyllene, α-pinene, transcriptome, functional differentiation

## Abstract

Red bayberry is a sweet, tart fruit native to China and grown widely in the south. The key organic compounds forming the distinctive aroma in red bayberry, are terpenoids, mainly β-caryophyllene and α-pinene. However, the key genes responsible for different terpenoids are still unknown. Here, transcriptome analysis on samples from four cultivars, during fruit development, with different terpenoid production, provided candidate genes for volatile organic compound (VOC) production. Terpene synthases (TPS) are key enzymes regulating terpenoid biosynthesis, and 34 TPS family members were identified in the red bayberry genome. *MrTPS3* in chromosome 2 and *MrTPS20* in chromosome 7 were identified as key genes regulating β-caryophyllene and α-pinene synthesis, respectively, by qRT-PCR. Subcellular localization and enzyme activity assay showed that MrTPS3 was responsible for β-caryophyllene (sesquiterpenes) production and MrTPS20 for α-pinene (monoterpenes). Notably, one amino acid substitution between dark color cultivars and light color cultivars resulted in the loss of function of MrTPS3, causing the different β-caryophyllene production. Our results lay the foundation to study volatile organic compounds (VOCs) in red bayberry and provide potential genes for molecular breeding.

## Introduction

Red bayberry (*Morella rubra*, formerly *Myrica rubra*), an evergreen plant belonging to Myricaceae, Fagales, is one of the most economically important fruits in southern China, with a production of over one million tons each year. It is also distributed in Korea, Japan, Southeast Asia and Australia (Chen et al., 2003). It is rich in anthocyanins, proanthocyanidins, vitamin C, and volatile organic compounds (VOCs), and is known as the “treasure fruit of Jiangnan,” highly appreciated by the vast number of consumers. There are over 100 cultivars or varieties in this species (Wu et al., 2020). Normally, the cultivar is identified by the color and size of the fruit. The dark color fruit cultivars, such as ‘Biqi’ (in the ‘Biqi’ series) and ‘Dongkui’ (in the ‘Dongkui’ series) contain the highest level of anthocyanin while the light color varieties, including ‘Xiazhihong’ and ‘Y2012-145’ (both belong to the ‘Fenhong’ series) have the lowest level of anthocyanin (Jiao et al., 2012; Jia et al., 2014). VOCs are mainly secondary metabolites, with terpenoids being a major part of them. Cheng et al. (2016) identified the component of VOCs and their content in 11 cultivars of red bayberry fruit by headspace solid-phase extraction. They found that the main aroma components in red bayberry were β-pinene, α-pinene (monoterpenes), D-limonene and β-caryophyllene (sesquiterpenes), with different cultivars having different kinds of terpenoids (Cheng et al., 2016).

The main products of the terpenoid pathway are monoterpene (C10), sesquiterpene (C15), and diterpene (C20). There are two conserved pathways for the biosynthesis of plant terpenoids: the mevalonate (MVA) and the methyl erythritol phosphate (MEP) pathway. The MVA pathway produces farnesyl diphosphate (FPP) that can be transformed to sesquiterpene on catalysis with sesquiterpene synthase in the cytoplasm while the MEP pathway produces geranyl diphosphate (GPP) that is catalyzed by monoterpene synthase to monoterpenes in plastids (Enfissi et al., 2005; Wang et al., 2018; Zhou and Pichersky, 2020).

Terpene synthases (TPS), also known as cyclases, are key enzymes for terpenoid biosynthesis. TPS family members are divided into seven subfamilies, TPS-a, TPS-b, TPS-c, TPS-d, TPS-e/f, TPS-g, and TPS-h (Chen et al., 2011). TPS-a, TPS-b, and TPS-g are unique to angiosperms. TPS-a mainly synthesizes sesquiterpenes and TPS-b mainly monoterpenes, while TPS-g can synthesize monoterpenes, sesquiterpenes and diterpenes (Chen et al., 2011). TPS family members could improve the aroma quality and resistance in fruit. In tomato, the content of linalool in mature fruit could be increased by introducing a linalool synthase under the control of the tomato late-ripening-specific E8 promoter (Lewinsohn et al., 2001). Similarly, the content of geraniol and citronellol in tomato was significantly increased while expressing the *Ocimum basilicum* geraniol synthase gene under the control of the tomato ripening-specific polygalacturonase promoter (Davidovich-Rikanati et al., 2007). Limonene is the volatile substance in the oil cell layer of citrus fruit, and the inhibition of limonene synthase has been shown to enhance their resistance to *Penicillium* (Rodríguez et al., 2014). UV-B treatment resulted in the downregulation of *PpTPS1* and upregulation of *PpTPS2*, causing a reduction in linalool and an increase in (E,E)-α-farnesene in peach, respectively (Liu et al., 2017). Transcription factors, including MYB, NAC, WRKY, EIN3, have been reported to regulate *TPS* genes, involved in terpenoid biosynthesis in many species (Nieuwenhuizen et al., 2015; Li et al., 2017; Jian et al., 2019). In two kiwifruit species with different terpenoid production (*Actinida arguta* and *A. chinensis*), AaNAC2, AaNAC3, and AaNAC4 were found to bind to the *AaTPS1* promoter but not to the *AcTPS1* promoter because of one SNP at the NAC binding site, leading to lower expression of *AcTPS1* and lower terpenoid content in *A. chinensis* (Nieuwenhuizen et al., 2015). However, the mechanism of terpenoid biosynthesis is still unclear, as is which TPS protein is responsible for the major aroma formation in red bayberry.

Here, red bayberry mainly produced β-caryophyllene or α-pinene in the studied four cultivars, so comprehensive RNA-seq analysis during fruit development was used and candidate genes screened for the accumulation of β-caryophyllene and α-pinene, respectively. The TPS gene family was extensively analyzed and two key TPS proteins, MrTPS3 and MrTPS20, were functionally characterized for aroma production in red bayberry.

## Materials and Methods

### Plant Materials

Fruit samples of ‘Biqi,’ ‘Dongkui,’ ‘Xiazhihong,’ and ‘Y2012-145’ cultivars were collected for RNA-seq at 44 days (S1), 54 days (S2), and 66 days (S3) after full blooming, in 2015, based on the fruit shape and color according to previous observations, and were immediately frozen in liquid nitrogen. Ten fruits were grouped as one biological replicate and stored at −80°C until use. In addition, 30 fruits were collected separately to determine physiological indexes of fruit quality such as single fruit weight, anthocyanins, soluble sugars, and soluble organic acids. Samples for qRT-PCR and gene cloning were collected in 2017 at the same stages as in 2015.

### Measurement of Soluble Sugars and Organic Acids

High performance liquid chromatography (HPLC) was used to determine soluble sugars and organic acids in fruit. The soluble sugars and organic acids were extracted from three gram of fruit powder in a 10 ml tube with 6 ml 80% ethanol, at 35°C in a water bath for 20 min, followed by centrifugation at 6,500 rpm for 15 min. The supernatant was collected while the pellet was resuspended in another 6 ml of 80% ethanol, and the extraction repeated three times. The supernatant mixture was diluted to 25 ml with 80% ethanol. One ml of extracts was dried by vacuum concentration at 45°C for 4 h. The crystal was dissolved with 1 ml ultrapure water and the solution centrifuged at 12,000 rpm for 15 min. The resulting supernatant was filtered through a 0.22 μM filtration membrane into sample vials for HPLC detection. The glucose, fructose, sucrose, and citric acid standards were from Sangon Biotech Co., Ltd. (Shanghai, China) and Shanghai Hushi Laboratorial Equipment Co., Ltd. (Shanghai, China).

The HPLC conditions for determination of soluble sugars were as follows: The mobile phase was acetonitrile: ultrapure water = 9:1 (v:v), with a flow rate of 1 ml min^–1^. The sample injection volume was 10 μl and the column temperature was set at 30°C. The Waters Spherisorb NH_2_ column was used for the separation and the RID detector (Waters, United States) was used for the detection.

The HPLC conditions to determine the organic acids were as follows: The mobile phase was 0.01 M (NH_4_)_2_HPO_4_: methanol = 97:3 (v:v), with a flow rate of 1 ml min^–1^. The sample injection volume was 10 μl and the column temperature was set at 35°C. The Waters SunFire C18 column was used for the separation and the SPD-M20A VWD detector (Agilent, United States) was used for detection.

### Measurement of Anthocyanins in Fruits

The measurement of anthocyanins was as previously described (Bai et al., 2019). In brief, 1 g of fruit powder was added to 5 ml of precooled HCl-methanol solution (1:999, v:v) for 24 h at 4°C in the dark, then centrifuged at 12,000 rpm for 20 min at 4°C. The anthocyanin was measured at 510 nm and 700 nm absorbance using a UV-VIS spectrophotometer.

### Measurement of Volatile Organic Compounds in Red Bayberry Fruits

The VOCs were extracted following the method previously described (Zhang et al., 2018). In brief, 1 g fruit samples was added to 2 ml of 20% NaCl (m/v) solution and mixed by vortex. 20 μl of 2-octanol (0.766 μg μl^–1^), as an internal standard, and 300 μl of CH_2_Cl_2_ was added and vortexed thoroughly. After 20 min incubation at room temperature, samples were centrifuged at 10,000 rpm for 5 min, and the supernatant transferred to a 1.5 ml centrifuge tube, to which 50 mg anhydrous sodium sulfate was added, and incubated for a further 30 min until the water was fully absorbed. For detection, 150 μl of the solution were transferred to GC vials, in a gas chromatography–mass spectrometry (GC-MS) (Agilent, 7890a, United States) equipped with a CTC-PAL2 (United States) autosampler.

### RNA Isolation and RNA Sequencing

RNA isolation and sequencing were as described previously (Jia et al., 2019). A modified cetyltrimethylammonium bromide method was used for RNA extraction. The RNA-seq library was constructed and sequenced by 1 gene Co., Ltd. (Hangzhou, China) on Illumina Hiseq 4000 platform with pair-end sequence (PE150). The raw data were generated and processed in a quality control step using FastQC and Trim-galore to trim the adaptors and low quality reads to produce clean data. Per sample was an average of 7.43 giga bases (Gb) of clean data.

### RNA-Seq Analysis

Next-generation sequencing clean reads were mapped to the red bayberry genome (Jia et al., 2019) using HISAT2 (Kim et al., 2019). Additionally, SAMtools (Danecek et al., 2021) was used to convert SAM files to BAM files. The reads covering transcripts were counted with featureCounts (Liao et al., 2014) and then used to identify DEGs with DEseq2. Genes with a false discovery rate (FDR) < 0.05, *P* < 0.05 and | log2Ratio| ≥ 1 were designated as DEGs, and the FPKM value for each gene was calculated. Heatmaps (scaled by row) were prepared using TBtools (Chen C. et al., 2020), and the R packages were used for Mfuzz and WGCNA analysis (Kumar and Futschik, 2007; Langfelder and Horvath, 2008).

### First Strand cDNA Synthesis and Quantitative Real-Time PCR

Samples for qRT-PCR were collected in 2017 as described above. First strand cDNA synthesis was with 1 μg total RNA with the HiScript II Q RT SuperMix for qPCR (+gDNA wiper) (Vazyme, China), and the qRT-PCR assay was completed with the iTaq™ Universal SYBR^®^ Green Supermix (Bio-Rad, United States) as described by Yang et al. (2020). The *MrPP2A* (KAB1212620.1), homologous to *AtPP2A*, was selected from the RNA-seq data and used as a reference control because it is constitutively expressed during fruit development in different cultivars and the primer efficiency is 96.6%. The qRT-PCR primers are listed in Supplementary Table 1.

### Identification, Phylogenetic Analysis, Conserved Motifs, and *Cis*-Element Prediction of Terpene Synthases Genes

A hidden Markov model (HMM) profile was constructed with the Terpene synthase N terminal domain (PF01397) and Terpene synthase C terminal domain (PF03936) from the Pfam database^1^ (El-Gebali et al., 2018). An HMM search was carried out in red bayberry protein databases using the HMM profiles that had been constructed. Protein sequences encoded by the TPS family genes were used as query with the HMMER 3.0 software package (Finn et al., 2015). After manually refining incorrect predicted genes, 34 MrTPS proteins were identified. Chromosomal locations of the red bayberry TPS genes were obtained based on the red bayberry genome information (Jia et al., 2019).

Phylogenetic and molecular evolutionary analyses were using MEGA 7.0 (Kumar et al., 2016). Red bayberry TPS protein sequences identified above were aligned with TPSs from other species using ClustalW with the default settings in MEGA 7.0. The Neighbor-Joining method in MEGA was used to construct the phylogenetic trees with 1,000 bootstrap replicates as previously described (Yang et al., 2019). To identify shared motifs and structural divergences among the predicted TPS family proteins, the MEME online tool^2^ was used with the following parameters: maximum number of motifs, 6; minimum motif width, 10; and maximum motif width, 60. TBtools (Chen C. et al., 2020) was used for motif visualization and gene structure analysis. Putative promoters of *TPS* genes, which were 2,500 bp upstream of the transcription start site or start codon, were extracted from the genome sequence (Jia et al., 2019). About 2,400 and 1,350 bp were cloned for proMrTPS3 and proMrTPS20, respectively. *Cis*-elements were predicted using an online tool PlantCARE (Lescot et al., 2002) and visualized using TBtools (Chen C. et al., 2020).

### Subcellular Localization Assay and 3D Protein Model Prediction

*Agrobacterium tumefaciens* GV3101 were transformed with the 35S:MrTPS3-GFP, 35S:MrTPS20-GFP, and 35S:GFP (free GFP) constructs. The encoded proteins and AtRUBISCO-RFP, which was used as a chloroplast marker, were co-expressed in transgenic tobacco leaves as previously described (Yang et al., 2018). In detail, the positive clones were picked and cultured in 5 ml liquid LB medium (containing 50 mg/L hygromycin and 50 mg/L rifampicin) at 28^°^C overnight with shaking. The agrobacteria were centrifuged at 5,000 rpm for 5 min and resuspended with an infection solution [containing 10 mM MgCl_2_, 10 mM MES (pH = 5.6), and 100 μM acetosyringone] to adjust the OD_600_ to 1.0. Agrobacteria harboring GFP constructs were mixed in equal volume with the agrobacteria harboring AtRUBISCO-RFP. After 1 h at room temperature, the infection solution was injected with a 1 ml syringe into the back of the 4th–6th leaves of tobacco. The tobacco plants were incubated at 25°C (day)/23°C (night) for 36–48 h, and fluorescence detected with a laser confocal microscope (Nikon, Japan). Primers used for constructing plasmids are listed in Supplementary Table 2.

Protein 3D models were predicted using the online tool SWISS-MODEL.^3^ The full length of MrTPS3 and MrTPS20 proteins were uploaded using the sequences obtained from cloning.

### Prokaryotic Protein Expression and Enzyme Activity Assay

The full CDS of *MrTPS3* was cloned into pET-32a and the protein was expressed in *Escherichia coli* strain BL21 (Rosetta) and purified using Ni-NTA Sefinose Resin (Settled Resin) (Sangon Biotech, China) and Econo-Pac^®^ Disposable Chromatography Columns (Bio-Rad) as previously described (Yang et al., 2020). The purified His-MrTPS3 protein was dialyzed with 1M Tris-HCl [containing 10% glycol and 20 mM dithiothreitol (DTT)] solution. The enzyme activity assay was carried out according to methods described in Liu et al. (2017). Primers used for constructing plasmids are listed in Supplementary Table 2.

## Results

### Fruit Development and Major Volatile Organic Compounds in Cultivars of Red Bayberry

**FIGURE 1:**
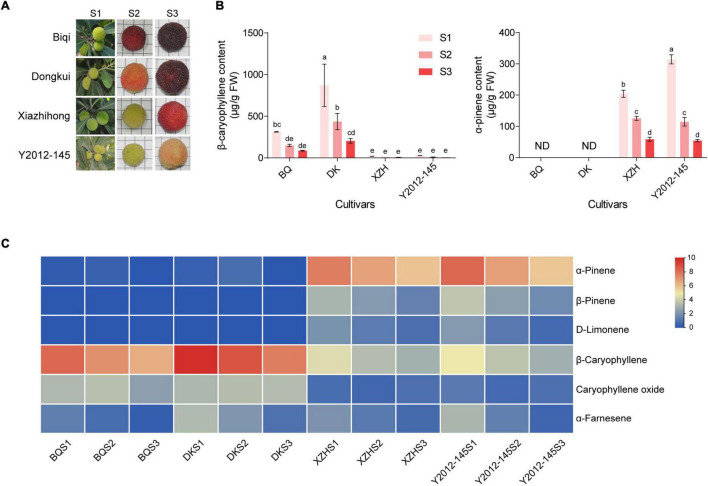
Fruit appearance and VOC contents in four cultivars of red bayberry. **(A)** Photos of red bayberry fruits at three stages in four cultivars. S1, S2, S3 show the three fruit developmental stages. **(B)** Main VOCs in red bayberry. “ND” indicates not detectable. “BQ,” “DK,” and “XZH” indicate ‘Biqi,’ ‘Dongkui,’ and ‘Xiazhihong’ cultivars, respectively. Error bars indicate standard deviation of three biological replicates. Different letters indicate significant difference among stages and cultivars (one-way ANOVA, *p* < 0.05). **(C)** Heatmap of VOC contents at three development stages in the four cultivars. Normalized contents are showed in blue (low) to red (high).

The fruits of four red bayberry cultivars (‘Biqi,’ ‘Dongkui,’ ‘Xiazhihong,’ and ‘Y2012-145’) were collected at three developmental stages (Figure 1A). The most obvious differences among the four cultivars were fruit size and color. The fruit of ‘Biqi’ was small (mean weight per fruit was 9.86 g), with the darkest color and the highest anthocyanin content (Supplementary Figures 1A,B). The fruit of ‘Xiazhihong,’ medium in size (mean weight per fruit was 13.73 g), was a lighter color and had lower anthocyanin content while the fruit of ‘Y2012-145,’ also medium in size (mean weight per fruit was 12.23 g), was the lightest color and had the lowest anthocyanin content (Supplementary Figures 1A,B). The fruit of ‘Dongkui’ was the largest in size (mean weight per fruit was 20.08 g) with high anthocyanin content (Supplementary Figures 1A,B). The single fruit weight of fruit at the S2 to S3 developmental stage suggested that the weight of ‘Biqi’ (small fruit cultivar) changed little, while that of ‘Dongkui’ more than doubled (Supplementary Figure 1A). The content of three soluble sugars, sucrose, fructose, and glucose, increased continuously during fruit development in the four cultivars (Supplementary Figure 1C), while the content of citric acid, the most abundant organic acid in red bayberry fruits, gradually decreased (Supplementary Figure 1D), during the maturation of fruits.

As the main VOCs in red bayberry differ between cultivars (Cheng et al., 2016), the VOCs in fruits of the four cultivars was measured using GC-MS. The results showed that the fruits with high anthocyanin contents (‘Biqi’ and ‘Dongkui’) mainly produced β-caryophyllene while the fruits with low anthocyanin contents (‘Xiazhihong’ and ‘Y2012-145’) mainly produced α-pinene (Figures 1B,C). In addition, both β-caryophyllene and α-pinene decreased with fruit development, with the highest concentration at the S1 stage (Figures 1B,C). Notably, in ‘Biqi’ and ‘Dongkui’ fruit, α-pinene, β-pinene, and D-limonene were almost not detectable (Figure 1C).

### Changes in Transcript Levels and Genes Related to Volatile Organic Compound Biosynthesis

To investigate the mechanism of VOC biosynthesis in red bayberry, RNA sequencing was used to reveal the different biosynthetic mechanisms of β-caryophyllene and α-pinene in red bayberry. A total of 267.57 G bases of clean data were obtained with 36 samples (Supplementary Table 3). Reads were mapped to the reference genome (Jia et al., 2019), with a mapping rate of more than 75% for all samples (Supplementary Table 3). A total of 26,809 DEGs were identified according to the conditions of *P* < 0.05, FDR < 0.05 and | log2Ratio| ≥ 1. Statistical analysis of DEGs showed that in the transition from S1 to S2, the number of up-regulated genes was more than that of down-regulated genes in four cultivars (Supplementary Table 4).

**FIGURE 2:**
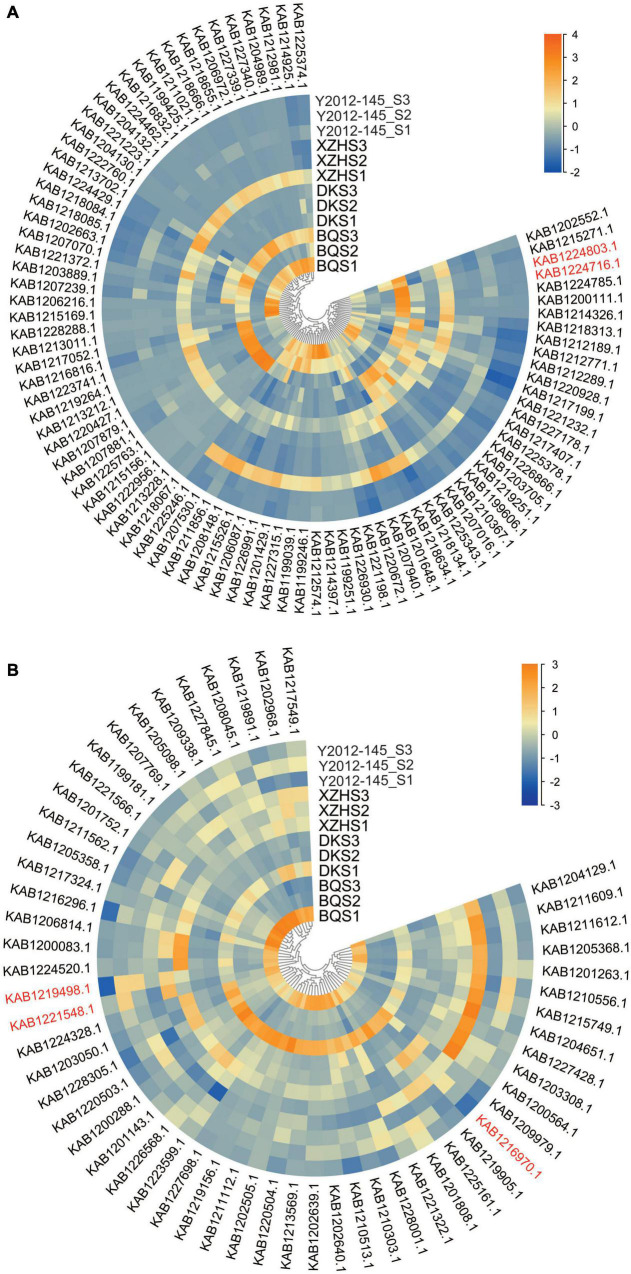
Expression profiles of the selected gene set. **(A)** Expression files of genes that were specifically downregulated in ‘Biqi’ and ‘Dongkui.’ **(B)** Expression files of genes that were specifically downregulated in ‘Xiazhihong’ and ‘Y2012-145.’ Gene IDs in red font indicate the genes mentioned in the main text. Genes were clustered according to their expression patterns.

For further clarification, the Mfuzz R package was used to cluster the expression patterns of the DEGs from ‘Biqi,’ ‘Dongkui,’ ‘Xiazhihong,’ and ‘Y2012-145’ at the three stages. Four expression clusters were obtained in each cultivar during the three developmental stages (Supplementary Figure 2). Cluster 1 in ‘Biqi,’ Cluster 2 and 3 in ‘Dongkui,’ Cluster 4 in ‘Xiazhihong’ and Cluster 2 in ‘Y2012-145’ showed continuous downregulation of gene expression (Supplementary Figure 2), consistent with the changes in the β-caryophyllene and α-pinene content, suggesting that some of these DEGs might be involved in β-caryophyllene or α-pinene biosynthesis. A Venn diagram was constructed and the genes shared in all of the groups and genes specific to ‘Biqi’ and ‘Dongkui’ or ‘Xiazhihong’ and ‘Y2012-145’ identified (Supplementary Figure 3). From the heatmap of the expression profiles of the gene set (Figure 2), two bHLHs (KAB1224803.1 and KAB1224716.1) genes showed continuous downregulation and the expression of the two bHLHs were higher in ‘Biqi’ and ‘Dongkui’ but lower in ‘Xiazhihong’ and ‘Y2012-145’ (Figure 2A). This suggests that these two bHLHs are candidate transcription factors regulating β-caryophyllene biosynthesis. The transcription factors WRKY22 (KAB1219498.1), ERF1A (KAB1216970.1), and BZR1 (KAB1221548.1) also had a specific expression pattern in ‘Biqi’ and ‘Dongkui’ (Figure 2B), suggesting the possible role of brassinosteroids in VOC biosynthesis.

**FIGURE 3:**
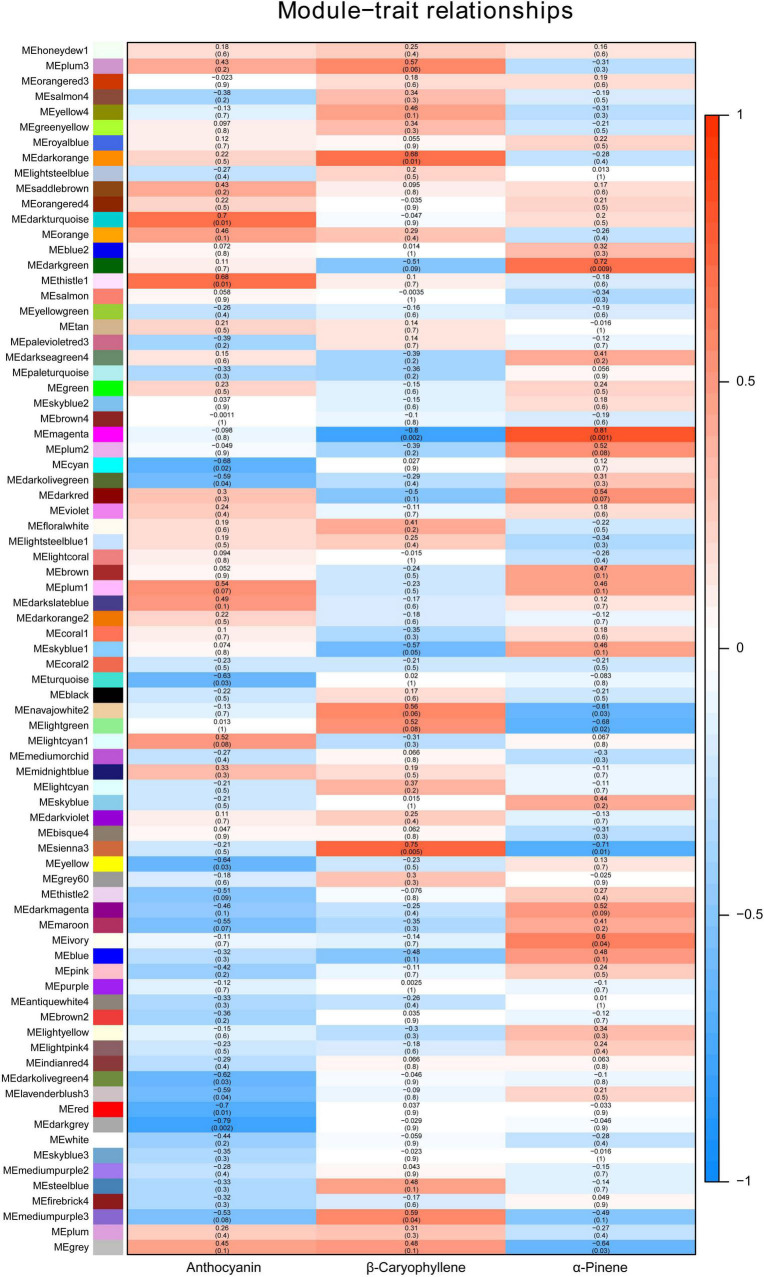
Module-trait relationship of WGCNA. Seventy-eight different modules were grouped for all expressed genes. Colors and numbers indicate the correlation coefficient between gene expressions and compound contents. Numbers in the brackets show the *p*-values of the significance.

Weighted gene co-expression network analysis (WGCNA) is an effective way to mine the genes related to a specific phenotype. WGCNA with all the genes obtained in the RNA-seq data gave a total of 78 modules, grouped according to the co-expression with the content of anthocyanin, β-caryophyllene and α-pinene (Figure 3). Interestingly, many modules showed the opposite correlations between β-caryophyllene and α-pinene content, including “MEdarkgreen,” “MEmagenta,” “MEnavajowhite2,” “MElightgreen,” and “MEsienna3” (Figure 3). The modules with the highest confidence level, “MEmagenta” (cor = 0.81, *p* = 0.002) and “MEsienna3” (cor = 0.75, *p* = 0.005), were chosen for further analysis. “Module membership vs. gene significance” analysis showed the linear correlation between gene expression and VOCs content, and genes with module membership higher than 0.9 were considered as candidate genes for β-caryophyllene and α-pinene accumulation (Supplementary Figures 4A,B and Supplementary Tables 5, 6). Among the 26 genes identified in “MEsienna3,” the expressions of CASP-like genes were higher in ‘Biqi’ and ‘Dongkui’ than in ‘Xiazhihong’ and ‘Y2012-145’ (Supplementary Table 5). Acid phosphatase 1-like (KAB1212067.1) had very high expression compared to other candidate genes and was also more highly expressed in ‘Biqi’ and ‘Dongkui’ (Supplementary Table 5), indicating the possible role of acid phosphatase 1-like in β-caryophyllene production. In “MEmagenta,” 100 genes were identified as candidate genes negatively related to β-caryophyllene production and positively related to α-pinene production (Supplementary Table 6).

As terpenoids are synthesized in the cytosol via the MVA pathway or in the plastid via the MEP pathway, the expression of the structural genes in both terpenoid biosynthetic pathways in the four cultivars was further checked. Structural genes were identified using the BLASTp program with proteins in Arabidopsis as query. However, there was no significant difference between these structural genes in the four cultivars at the three developmental stages (Supplementary Figure 5).

### Genome-Wide Identification of Terpene Synthases Genes in Red Bayberry

**FIGURE 4:**
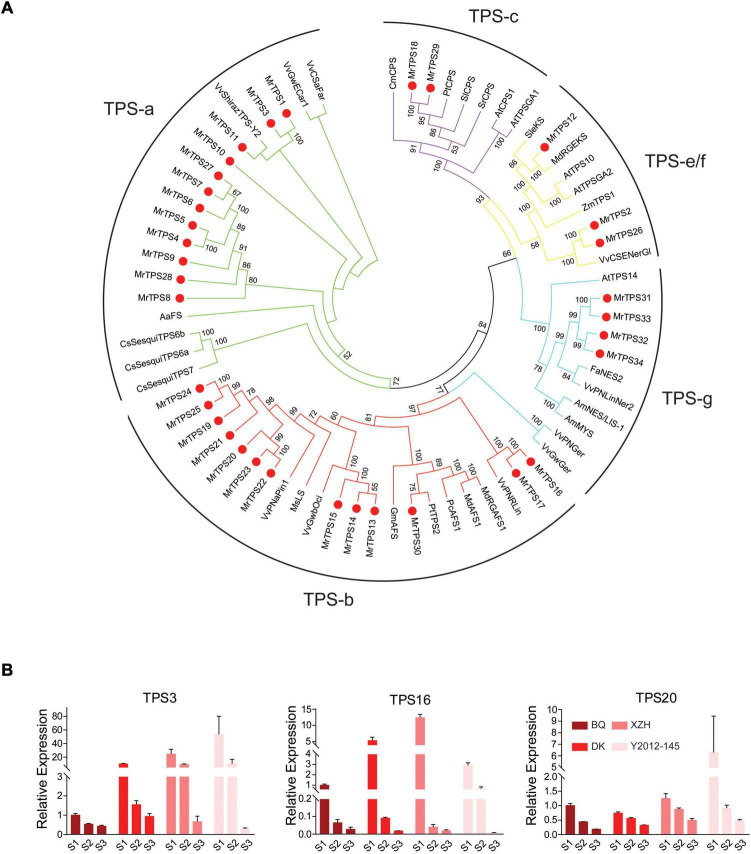
Phylogenetic analysis of TPSs and their expression patterns in different cultivars. **(A)** Phylogenetic tree of TPS family members in different species. Neighbor-Joining method in MEGA was used to construct the phylogenetic trees with 1,000 bootstrap replicates. Red dots indicate the TPS members in red bayberry. Lines with different colors show the different subfamilies. Bootstrap values higher than 50 are shown. **(B)** qRT-PCR analysis of the three selected candidate *TPS* genes. Error bars indicate standard deviation of three biological replicates. S1, S2, S3 show the three fruit developmental stages.

In order to clarify why the different types of VOCs were biosynthasized in the different cultivars, genome-wide identification of the terpenoid synthases family, the key enzymes for terpenoids production, was conducted. A total of 34 *MrTPS* genes were identified by searching Pfam domain in the red bayberry genome (Jia et al., 2019), named *MrTPS1*-*MrTPS34* according to the order of the TPSs location on the chromosomes (Supplementary Table 7). The *MrTPS* genes were distributed on six chromosomes, except chromosome 3 and 5 (Supplementary Table 7 and Supplementary Figure 6). *MrTPS4*-*MrTPS9* were tandemly located on chromosome 4, *MrTPS13*-*MrTPS17* on chromosome 6 and *MrTPS19*-*MrTPS23* were tandemly located on chromosome 7 (Supplementary Figure 6). The phylogenetic analysis of MrTPSs and TPSs in other species showed that 12 MrTPS proteins belonged to the TPS-a subfamily; 13 belonged to the TPS-b subfamily; two belonged to the TPS-c subfamily; three MrTPS belong to the TPS-e/f subfamily; four belong to the TPS-g subfamily (Figure 4A). No MrTPS proteins was found belonging to the TPS-d subfamily (Figure 4A).

To further identify the MrTPS family members, motifs were searched by the MEME tool. The results showed that most of the MrTPS proteins had motif 1, motif 2 and motif3, while MrTPS21 only had motif 3, identified as the key domain (DDxxD) for identification of most TPS family members (Supplementary Figure 7).

### Expressions and *Cis*-Element Analysis of MrTPS3 and MrTPS20

In order to screen the specific *MrTPS* genes that regulate the biosynthesis of α-pinene and β-caryophyllene in red bayberry fruits, the differentially expressed genes annotated as *TPS* by KEGG in the transcriptome data were analyzed. Based on the difference of the main VOCs in the four cultivars, the differentially expressed *MrTPS*s between ‘Biqi’ and ‘Xiazhihong,’ ‘Biqi’ and ‘Y2012-145,’ ‘Dongkui’ and ‘Xiazhihong,’ and ‘Dongkui’ and ‘Y2012-145’ were compared at the three developmental stages. 34 differentially expressed *TPS* genes were obtained at the S1 stage, 32 at the S2 stage, and only 21 at the S3 stage (Supplementary Figure 8). Among them, 15 *TPS* genes were obtained as DEGs at the three stages. By overlaping these genes with 34 *MrTPS* genes identified by Pfam domain and removing genes with no expression in the RNA-seq, six candidate *MrTPS* genes were taken out: *MrTPS1*, *MrTPS3*, *MrTPS10*, *MrTPS11*, *MrTPS16*, and *MrTPS20*. The expression of the six *MrTPS* genes were validated by qRT-PCR and the results showed that the expression of *MrTPS1*, *MrTPS10*, and *MrTPS11* were too low for qRT-PCR and cloning (Ct value > 35) in four cultivars at almost all stages. Only *MrTPS3*, *MrTPS16*, and *MrTPS20* had a high and stable expression in qRT-PCR in certain cultivars. As both *MrTPS16* and *MrTPS20* belonged to TPS-b subfamily and the expression of *MrTPS20* was much higher than that of *MrTPS16* in all the samples, the focus was on *MrTPS3* and *MrTPS20* for further analysis. Notably, the expression of *MrTPS20* was higher in ‘Xiazhihong’ and ‘Y2012-145’ than that in ‘Biqi’ and ‘Dongkui’ at all stages (Figure 4B). *MrTPS3*, which belonged to TPS-a, had much higher expression in ‘Xiazhihong’ and ‘Y2012-145.’

To explain the expression patterns of *MrTPS3* and *MrTPS20*, we cloned the promoter of *MrTPS3* and *MrTPS20* in the four cultivars. The results showed only SNPs difference in the promoter of *MrTPS3* and *MrTPS20*. For the *MrTPS3* promoter, *cis*-element analysis showed that ‘Biqi’ and ‘Dongkui’ had the same *cis*-element pattern while ‘Xiazhihong’ and ‘Y2012-145’ shared a different pattern with one more SA-responsive *cis*-element, indicating that salicylic acid might regulate the expression of *MrTPS3* (Supplementary Figure 9A). However, no consistent change of the *MrTPS20* promoter in the four cultivars was observed (Supplementary Figure 9B).

### Functional Characterization of MrTPS3 and MrTPS20

**FIGURE 5:**
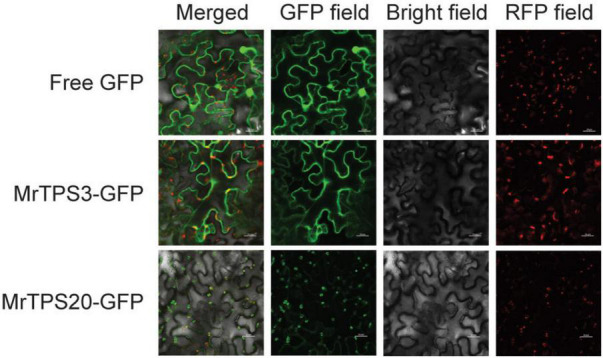
Subcellular localization of MrTPS3 and MrTPS20. Free GFP served as a positive control and Arabidopsis RUBISCOA-RFP served as a marker located in chloroplast. Bars indicate 50 μm.

**FIGURE 6:**
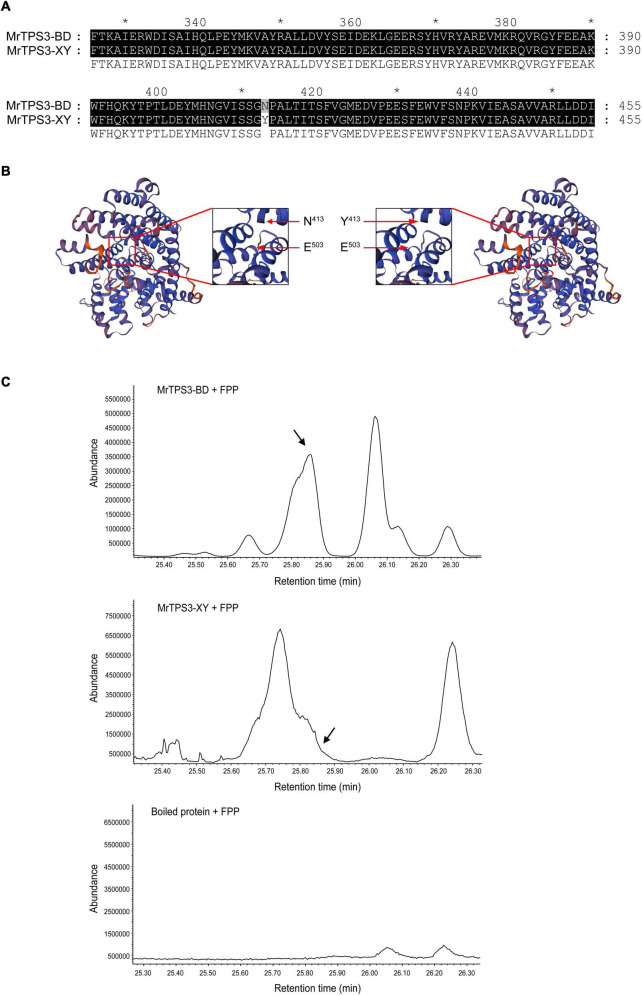
Functional characterization of MrTPS3. **(A)** Protein sequence alignment of MrTPS3 in ‘Biqi’/‘Dongkui’ (MrTPS3-BD) and in ‘Xiazhihong’/‘Y2012-145’ (MrTPS3-XY). **(B)** Protein 3D models for MrTPS3-BD and MrTPS3-XY. Pictures in the red box showed the zoom in of the picture. Arrows indicate the amino acid substitution and the structure change. **(C)** Enzyme activity assays of MrTPS3 using prokaryotic-expressed protein. GPP and FPP served as substrates and β-caryophyllene was the product. GC-MS was used to detect the reaction. Boiled MrTPS3 protein served as a negative control. The arrows show the peak of β-caryophyllene. The stars show the locations of amino acid sequence and the interval is 20 aa.

As α-pinene and β-caryophyllene are synthesized in different parts of a cell, the subcellular localization of MrTPS3 and MrTPS20 was verified. Transient expression of MrTPS3-GFP and MrTPS20-GFP in tobacco leaves showed that MrTPS3 was localized in the cytoplasm and nucleus while MrTPS20 was localized only in the chloroplast (Figure 5), indicating the different functions of these two TPS proteins. As β-caryophyllene is synthesized in cytoplasm and α-pinene is produced in chloroplasts, the results indicate that MrTPS3 is responsible for β-caryophyllene synthesis, while MrTPS20 produced α-pinene in red bayberry. Intriguingly, the CDS sequences of *MrTPS3* were the same in the dark color cultivars ‘Biqi’ and ‘Dongkui’ (MrTPS3-BD), and two SNPs were found in both light color cultivars ‘Xiazhihong’ and ‘Y2012-145’ (MrTPS3-XY) as compared to MrTPS3-BD (Supplementary Figure 10A). The similar phenomenon was observed in *MrTPS20*. Five SNPs were observed between MrTPS20-BD and MrTPS20-XY (Supplementary Figure 10B). The SNPs in MrTPS3 caused the asparagine (N^413^) to tyrosine (Y^413^) non-synonymous mutation (Figure 6A). The single amino acid substitution caused the conformational change at 503th glutamic acid (E^503^) in ‘Xiazhihong’ and ‘Y2012-145,’ leading to the change of the protein function (Figure 6B). The SNPs in MrTPS20 also resulted in single amino acid substitution (Supplementary Figure 10C), leading to the protein structure change (Supplementary Figure 10D), which might cause the loss of function of MrTPS20 in ‘Biqi’ and ‘Dongkui’ with no α-pinene production (Figure 1B).

To further confirm the functional differentiation of MrTPS3 between ‘Biqi’/‘Dongkui’ and ‘Xiazhihong’/‘Y2012-145,’ enzyme activity analysis of recombinant MrTPS3 proteins was conducted. The products were identified by GC-MS, which were compared to NIST libraries and authenticated compounds. The results showed that MrTPS3-BD could convert FPP to β-caryophyllene, while β-caryophyllene was not detected by MrTPS3-XY catalytic reaction (Figure 6B). Interestingly, MrTPS3-XY converted FPP to limonene (Supplementary Table 8) suggesting that the amino acid mutation in MrTPS3 changed the protein function. In addition, GPP was not recognized by MrTPS3 and there was no α-pinene production (Supplementary Figure 11). These results indicate that MrTPS3-BD is a sesquiterpene synthase for β-caryophyllene and the single amino acid substitution, N^413^ to Y^413^, results in the loss of function of MrTPS3-XY for β-caryophyllene production in red bayberry.

## Discussion

Aroma, which is mainly due to VOCs, is an important trait for fruit quality. Terpenoids are the most important VOCs in red bayberry and they are synthesized as a defense mechanism against pathogens and animals during fruit development (Zhou and Pichersky. 2020). TPS is considered as a key enzyme for VOCs production, and its identification and functional characterization would give a better understanding of its biosynthesis. With the publication of genomes of more and more species, the TPS family genes have been identified in many species in recent years. In citrus, a total of 55 *CsTPS* genes have been identified, and seven *TPS* genes are related to sesquiterpene biosynthesis, including one related to β-farnesene biosynthesis and two related to β–caryophyllene biosynthesis (Alquézar et al., 2017). More recently, 52 TPS family genes have been identified in wintersweet (*Chimonanthus praecox*), indicating a possible mechanism for the formation of flower aroma (Shang et al., 2020), while 100 *TPS* genes have been found in the lavender genome (Li et al., 2021). Here, the identification of 34 *TPS* genes in the red bayberry genome (Supplementary Table 7), much less than those in the genomes mentioned above, supports red bayberry not having had a whole genome duplication event (Jia et al., 2019). Similarly, in lily, 32 *LsTPS* genes have been identified, five of which are involved in the synthesis of three monoterpenes: ocimene, pinene, and limonene (Du et al., 2019).

Integrating the transcriptome and qRT-PCR data, *MrTPS3* and *MrTPS20* were identified as candidate genes for β-caryophyllene and α-pinene, respectively. SNPs in different cultivars often cause changes in protein functions, leading to different phenotypes. Intriguingly, ‘Biqi’ and ‘Dongkui’ have the same CDS sequences of *MrTPS3* while the sequences of *MrTPS20* are the same in ‘Xiazhihong’ and ‘Y2012-145,’ leading to a similar production of β-caryophyllene in ‘Biqi’ and ‘Dongkui’ and of α-pinene in ‘Xiazhihong’ and ‘Y2012-145.’ Our results showed that the single amino acid substitution from N^413^ to Y^413^ caused the loss of function of MrTPS3 (Figure 6), similar to the results from a study in rice, where one amino acid substitution from D to Y in TPSOg080 has been shown to cause the loss of activity of the TPS protein (Chen H. et al., 2020). The results obtained here confirmed that *MrTPS3* is the essential gene for β-caryophyllene production in ‘Biqi’ and ‘Dongkui.’ In a previous study, ‘Xiazhihong’ and ‘Y2012-145’ were found to have a very close relationship, both belonging to the ‘Fenhong’ series while the relationship between ‘Biqi’ and ‘Dongkui’ is more distant (Jia et al., 2014, 2015). Combining the results in this study, we clarified the close relationship between ‘Xiazhihong’ and ‘Y2012-145’ in aspects of aroma biosynthesis. As the offspring of the F1 population of ‘Biqi’ × ‘Dongkui’ have already produced fruits (Wang et al., 2020), further study could reveal the detailed mechanism of β-caryophyllene biosynthesis by using the genetic population. Meanwhile, other cross combinations could help to further characterize the differentiation of β-caryophyllene and α-pinene production by QTL mapping.

PpTPS2 in peach, belonging to the TPS-b subfamily and localized in the cytoplasm, has been induced by UV-B and participates in the biosynthesis of α-farnesene (Liu et al., 2017). Studies have shown that TPS genes are induced by JA and SA. Transcription factors involved in the JA and SA signaling pathways such as WRKY, MYC2, NAC, and MYB, participate in the regulation of expression of TPS genes in many plant species (Nieuwenhuizen et al., 2015; Li et al., 2017; Aslam et al., 2020). However, how TPS genes are transcriptionally regulated is still unclear in red bayberry. Here, WRKY22, ERF1A, and BZR1 were identified as candidate transcription factors regulating VOC biosynthesis (Figure 2). These transcription factors might regulate the expressions of *TPS* genes, with several related *cis*-elements (e.g., MBS, G-box, JA-responsive, and SA-responsive *cis*-elements) in the promoter of MrTPS3 and MrTPS20 identified (Supplementary Figure 9).

We observed that cultivars with high anthocyanin content mainly produced β-caryophyllene while cultivars with low anthocyanin content mainly produced α-pinene, which is consistent with a previous study (Cheng et al., 2016). Recent studies have shown the relationship between anthocyanin and terpenoids accumulation. In tomato, *SlMYB75* promotes anthocyanin accumulation and enhances VOCs production in the fruits, while overexpression of wintersweet *CpMYC2* causes higher linalool and β-caryophyllene production in Arabidopsis but less anthocyanin accumulation in transgenic tobacco flowers (Jian et al., 2019; Aslam et al., 2020). Further study is needed to verify the potential crosstalk between anthocyanin and terpenoid biosynthesis in red bayberry fruits as higher β-caryophyllene production was found to be related to the higher anthocyanin accumulation.

## Conclusion

**FIGURE 7:**
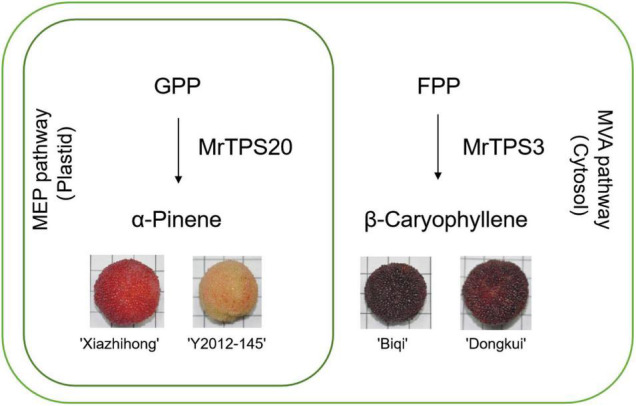
Summary of terpenoid biosynthesis in the four red bayberry cultivars. The outer green circle indicates a cell while the inner green circle indicates the plastid. ‘Xiazhihong’ and ‘Y2012-145’ mainly produce α-pinene in plastid, for which MrTPS20 is responsible. ‘Biqi’ and ‘Dongkui’ mainly produce β-caryophyllene in cytoplasm, for which MrTPS3 is responsible.

In conclusion, we obtained transcriptome data during development of fruit in red bayberry and carried out comprehensive analysis of the TPS gene family, identifying *MrTPS3* and *MrTPS20* as the central candidate genes for β-caryophyllene and α-pinene biosynthesis, respectively (Figure 7). One amino acid substitution causes the loss of function of *MrTPS3* in ‘Xiazhihong’ and ‘Y2012-145’ cultivars with lower β-caryophyllene production. These results lay the foundation for molecular biology studies during fruit development and give new insight into the molecular mechanism of VOC biosynthesis in red bayberry.

## Data Availability Statement

The data presented in the study are deposited in the NCBI repository (https://www.ncbi.nlm.nih.gov/), accession number PRJNA782750.

## Author Contributions

YW, ZG, and HMJ designed the experiment. YW and QY conducted most of the experiments and data analysis. YW, HMJ, YZ, LZ, PJ, and YJ collected the samples and carried out RNA-seq and qRT-PCR. YW, QY, YZ, and LZ conducted the prokaryotic protein expression and enzyme activity assay. YW and QY conducted the subcellular localization. HMJ, HJJ, GW, and CCZ collected the samples and conducted the fruit quality determination. YW, QY, and ZG wrote the manuscript. All authors contributed to the article and approved the submitted version.

## Conflict of Interest

The authors declare that the research was conducted in the absence of any commercial or financial relationships that could be construed as a potential conflict of interest.

## Publisher’s Note

All claims expressed in this article are solely those of the authors and do not necessarily represent those of their affiliated organizations, or those of the publisher, the editors and the reviewers. Any product that may be evaluated in this article, or claim that may be made by its manufacturer, is not guaranteed or endorsed by the publisher.

## Footnotes

1
https://pfam.xfam.org/

2
http://meme-suite.org/tools/meme

3
https://swissmodel.expasy.org/
