# Supplementary material for: MrTPS3 and MrTPS20 Are Responsible for β-Caryophyllene and α-Pinene Production, Respectively, in Red Bayberry (Morella rubra)
Source: Front Plant Sci. 2022 Jan 7;12:798086. doi: 10.3389/fpls.2021.798086 (PMC8777192; doi:10.3389/fpls.2021.798086)
Supplement: Supplementary file 1 [file Data_Sheet_1.PDF]

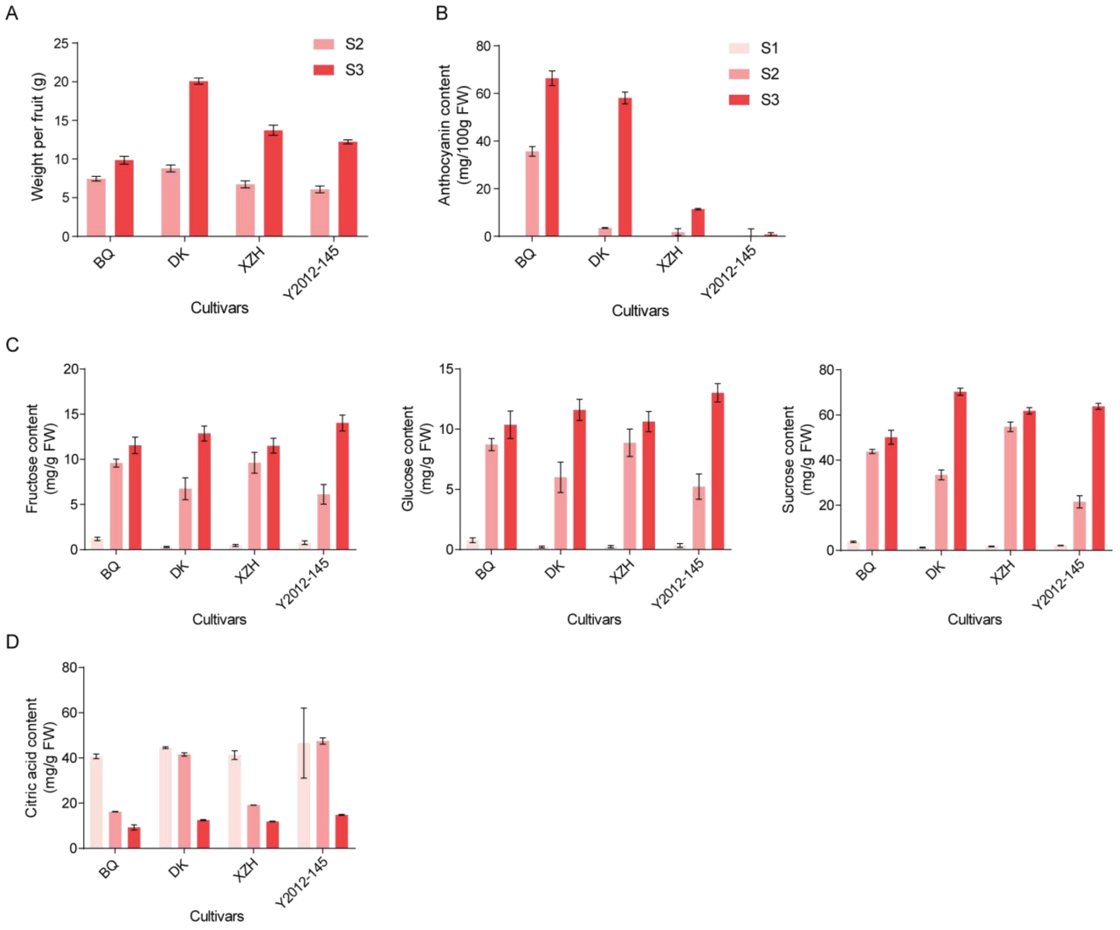

Fig. S1 Physiological indexes of red bayberry fruits. (A) Fruit weight of different cultivars of red bayberry at S2 and S3 stages. (B) Anthocyanin contents of different cultivars of red bayberry at the three stages. No anthocyanin was detected at S1 stage. (C) Soluble sugar contents of different cultivars of red bayberry at the three stages. (D) Contents of citric acids in different cultivars measured by HPLC. Error bars indicate standard deviation of three biological replicates. ‘BQ’ indicates ‘Biqi’ cultivar; ‘DK’ indicates ‘Dongkui’ cultivar; ‘XZH’ indicates ‘Xiazhihong’ cultivar.

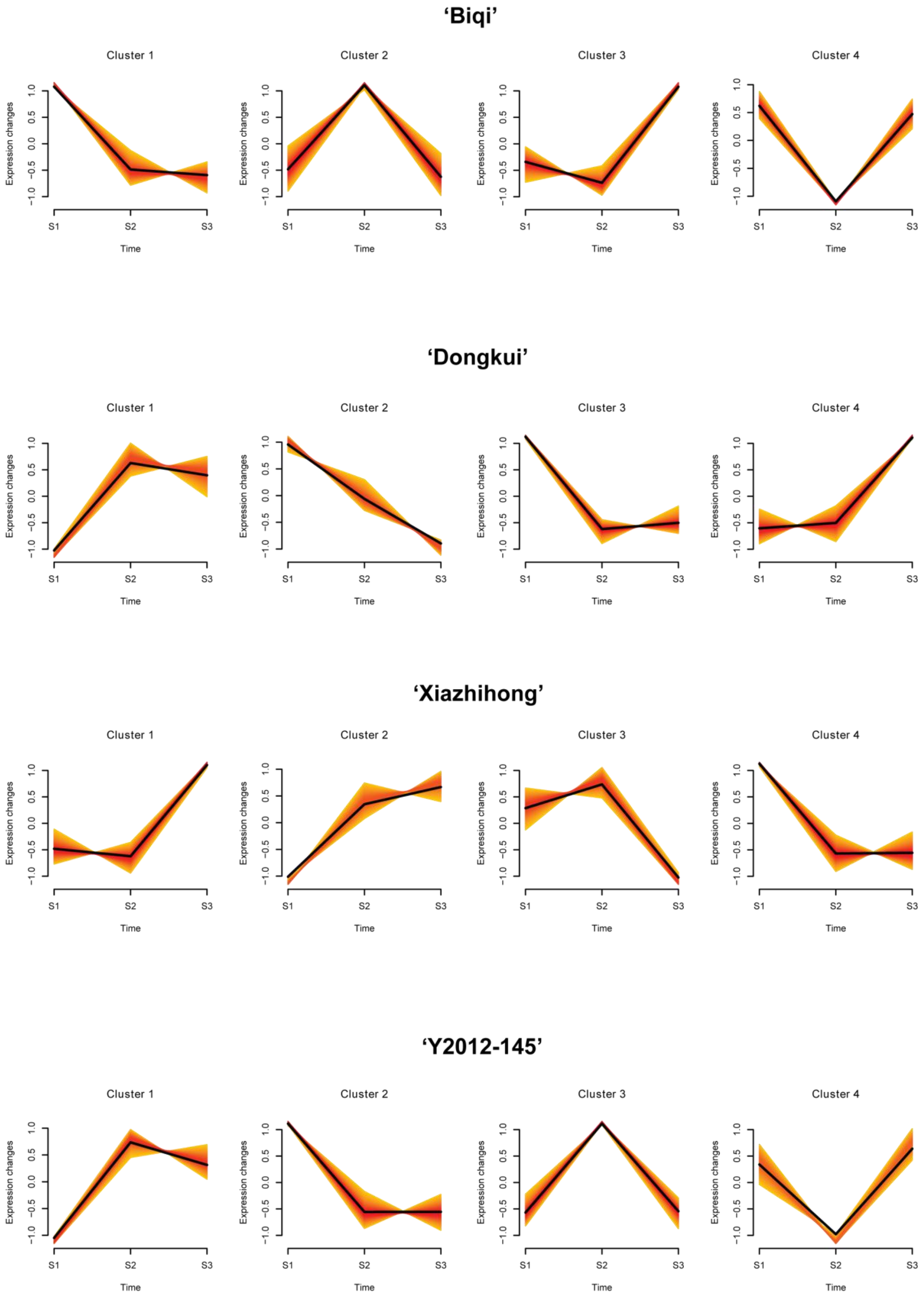

Fig. S2 Mfuzz clusters of DEGs in the four cultivars during the three stages of fruit development. Genes with membership over 0.5 were chosen. The black lines show the expression trends.

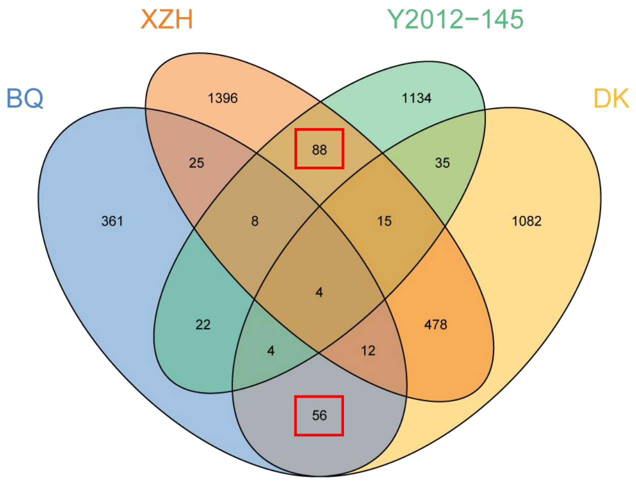

Fig. S3 Venn diagram of DEGs of the four cultivars from the chosen clusters in Mfuzz analysis. ‘BQ’ indicates DEGs in ‘Biqi’ cultivar; ‘DK’ indicates DEGs in ‘Dongkui’ cultivar; ‘XZH’ indicates DEGs in ‘Xiazhihong’ cultivar.

A

B

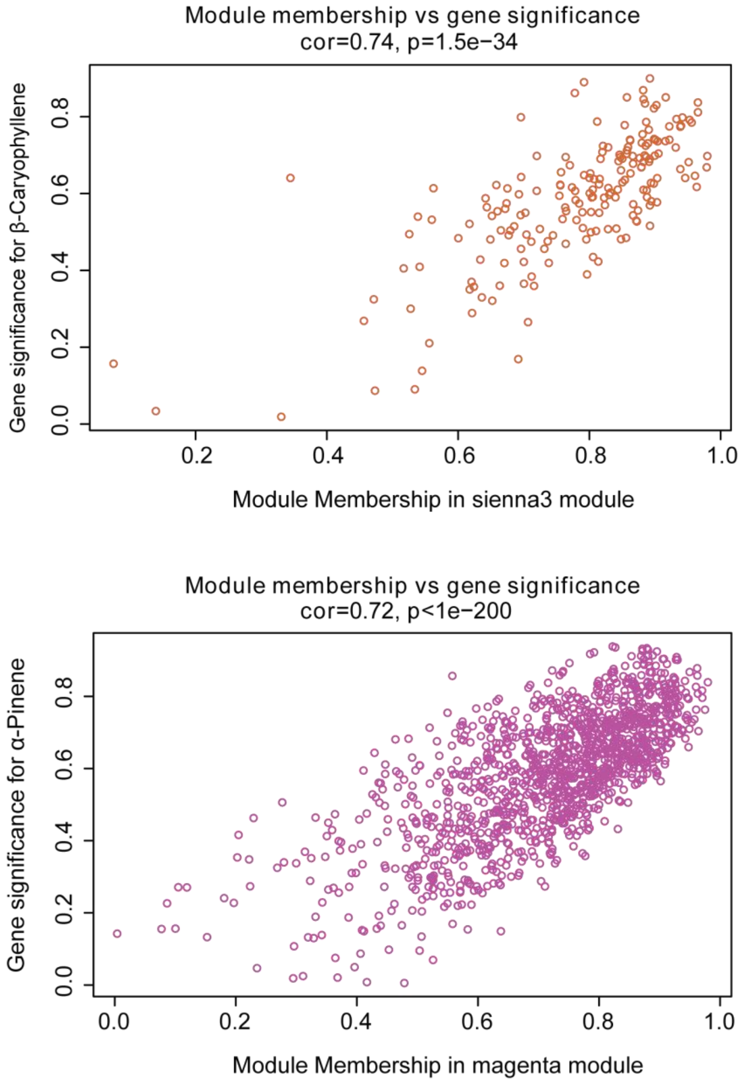

Fig. S4 Module memberships and gene significance in "MEsienna3" and "MEmagenta" modules in WGCNA analysis.

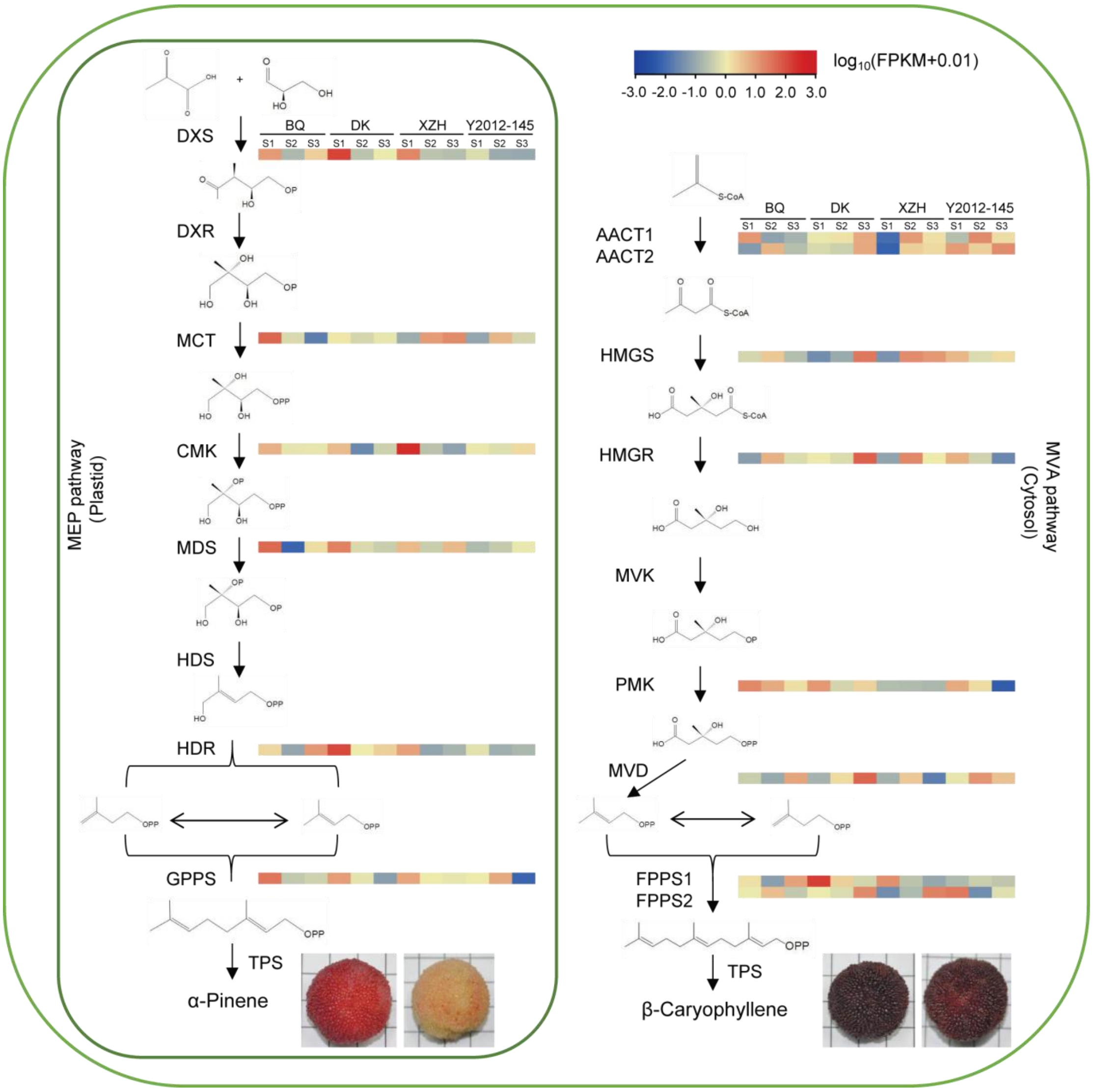

Fig. S5 Expression profiles of structural genes in the MEP and MVA pathways. MEP pathway is in plastid, which produces α-pinene while MVA pathway is in cytosol, which produces β-caryophyllene. The color bars show the expression level of the structural genes, which are normalized by log10(FPKM+0.01). The bars from left to right indicate the expression profiles in fruits of three stages in 'Biqi', 'Dongkui', 'Xiazhihong' and 'Y2012-145' respectively.

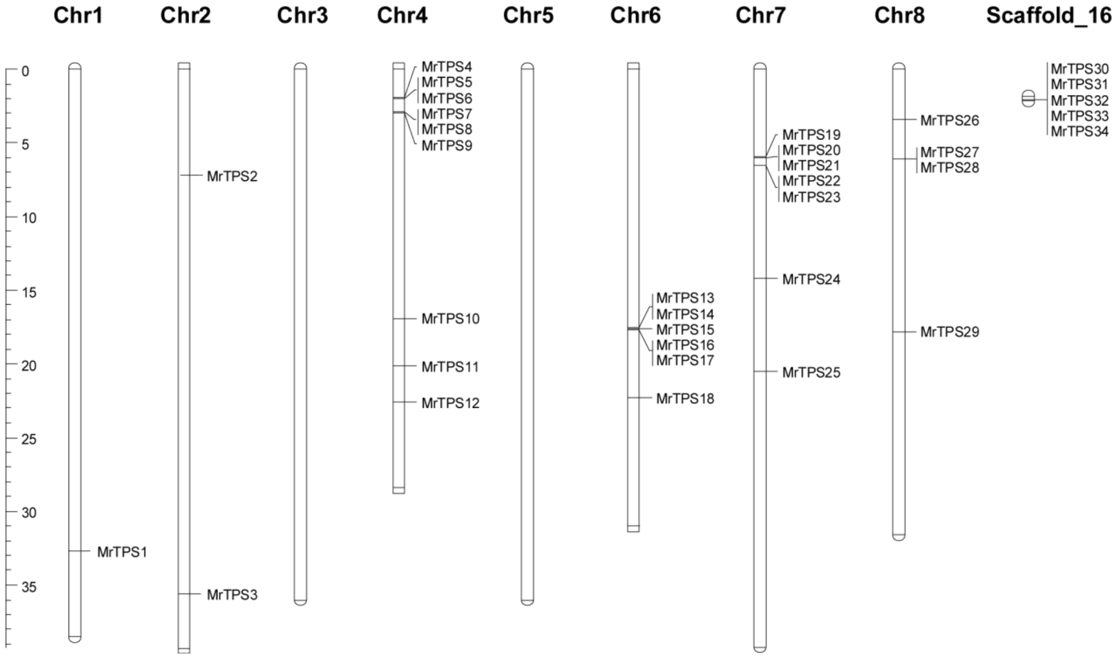

Fig. S6 The physical chromosome location of TPS genes in red bayberry. The bar shows physical distance. 29 TPS genes were anchored on 6 chromosomes while 5 were on the scaffold_16. Chr: chromosome.

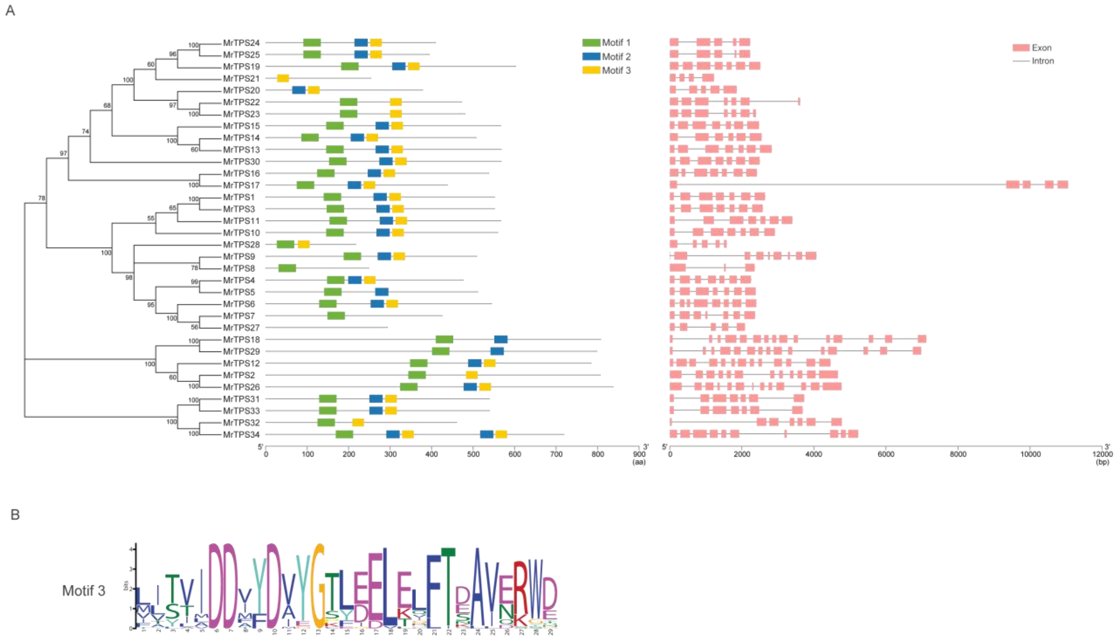

Fig. S7 Conserved motifs and gene structures of TPS family members in red bayberry. (A) Conserved motifs and gene structures. Boxes with different colors indicate different motifs or exons. (B) DDXXD motif of TPS proteins, which is essential for identification of most of the TPS proteins.

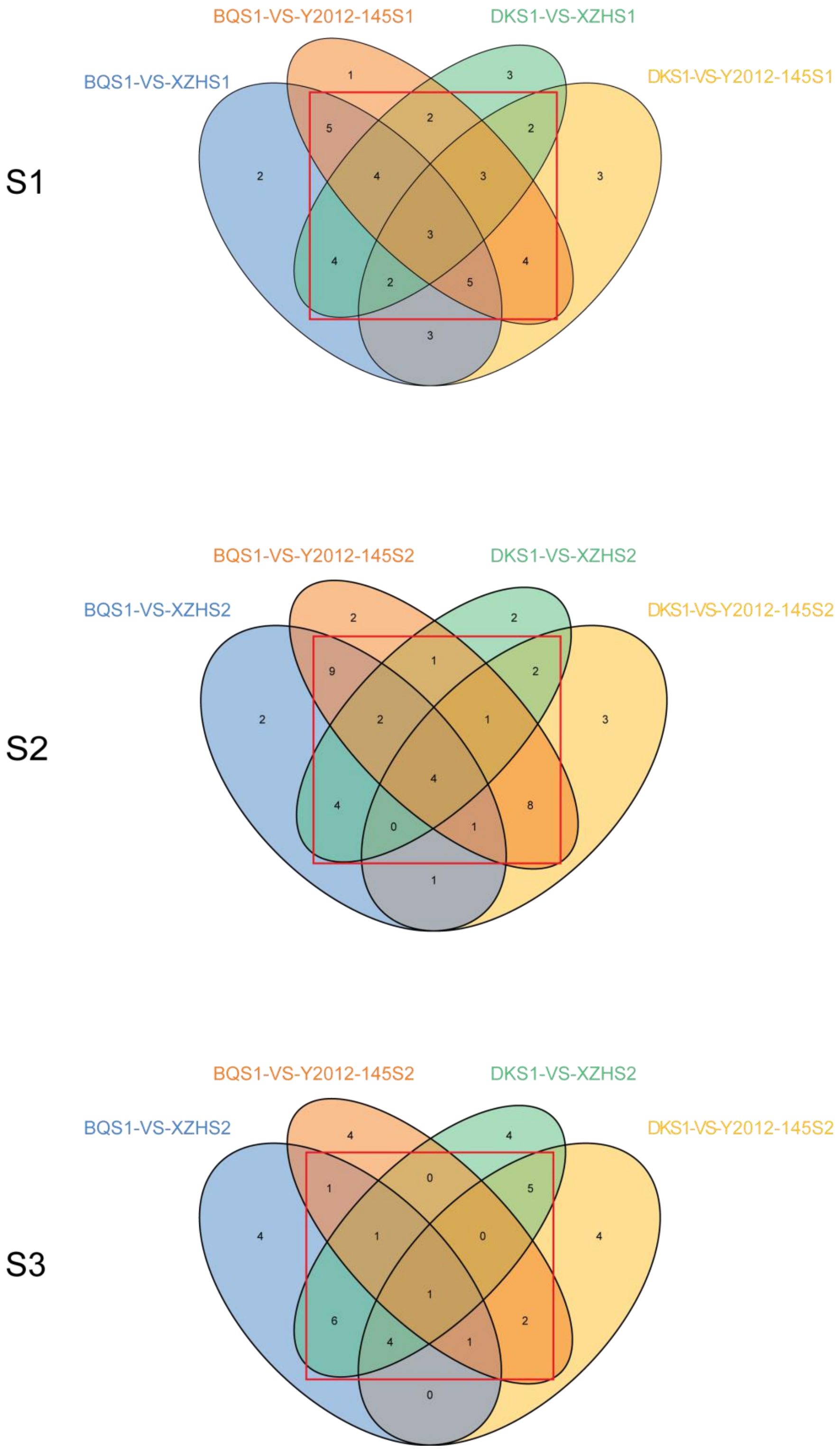

Fig. S8 Venn diagram of differentially expressed *TPS* genes in the red bayberry cultivars.Red boxes indicate candidate TPS genes with similar expression patterns in 'Biqi' (BQ) and 'Dongkui' (DK) or in 'Xiazhihong' (XZH) and 'Y2012-145'.

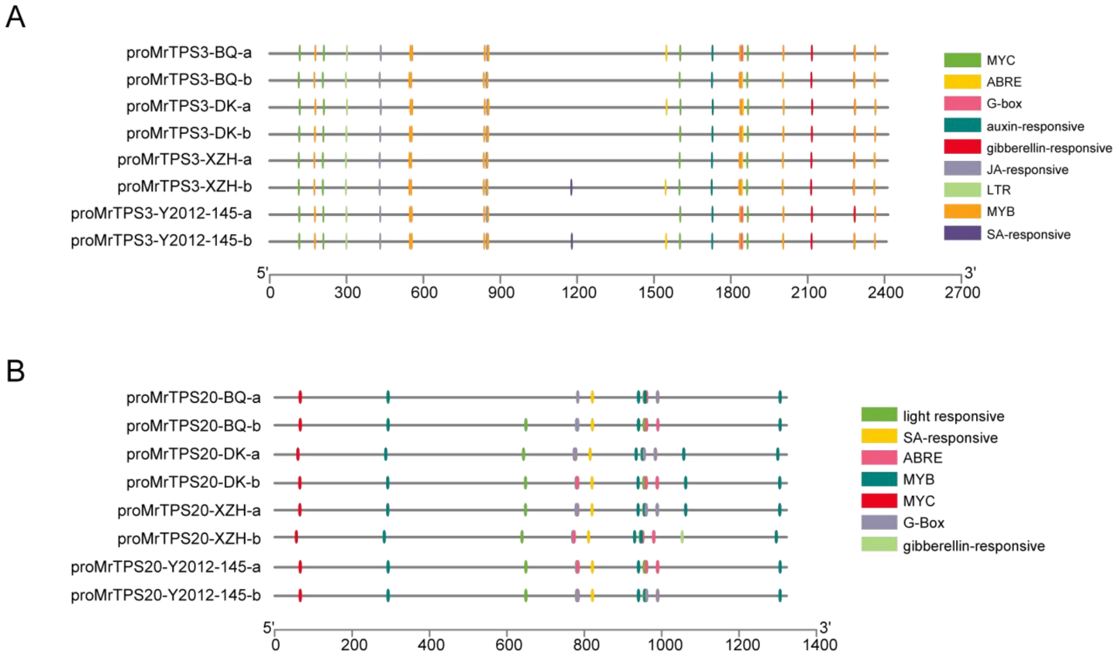

Fig. S9 Predicted *cis*-elements in the promoter of TPS3 and TPS20 in 'Biqi'/'Dongkui' and 'Xiazhihong'/'Y2012-145'. Boxes with different colors indicate different *cis*-elements. "-a" and "-b" indicate the allele in different cultivars.

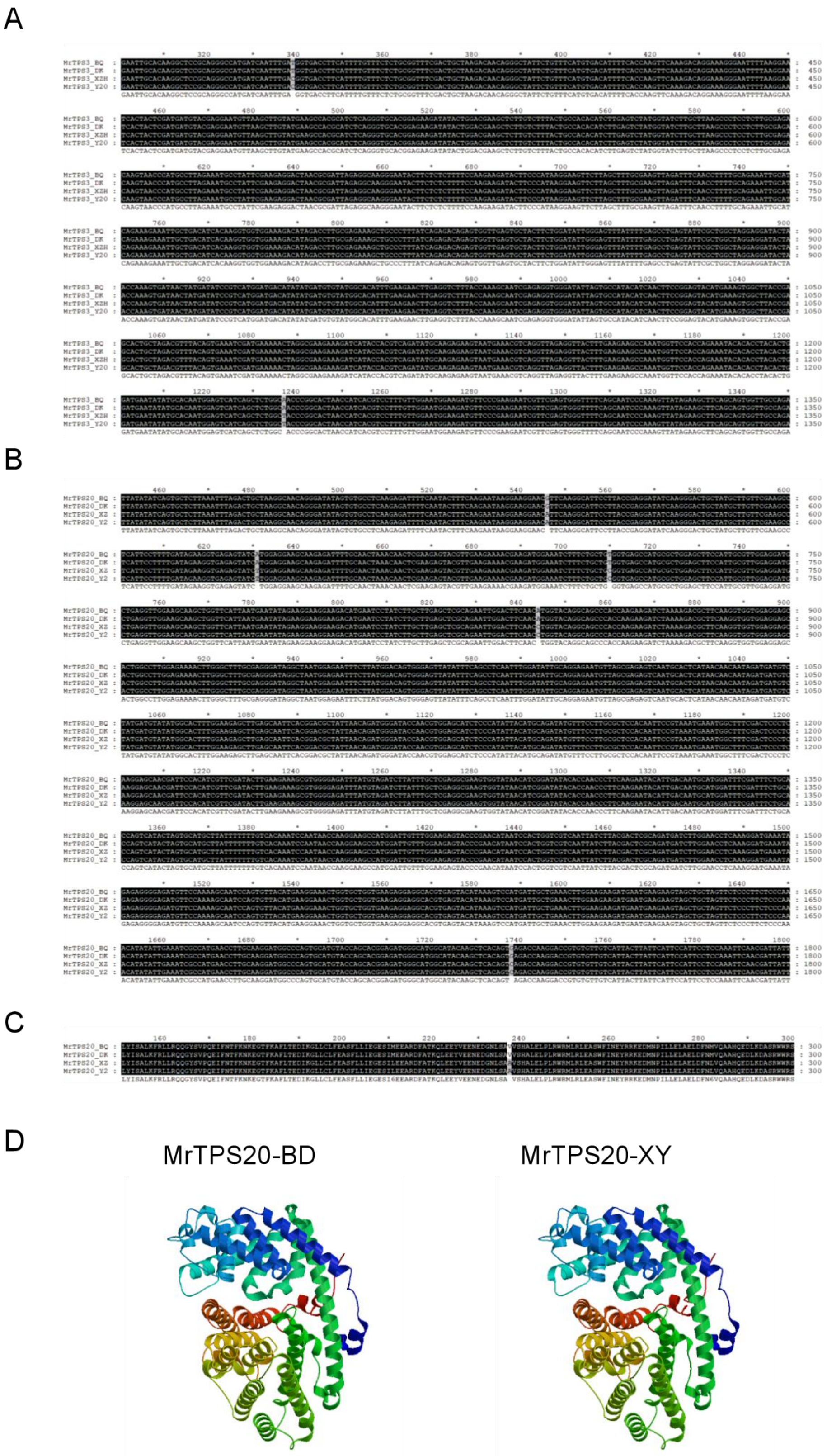

Fig. S10 Sequence alignment and protein 3D models. (A) Part of CDS sequence alignment of MrTPS3 in ‘Biqi’ (BQ), ‘Dongkui’ (DK), ‘Xiazhihong’ (XZH) and ‘Y2012-145’. (B) Part of CDS sequence alignment of MrTPS20 in ‘Biqi’ (BQ), ‘Dongkui’ (DK), ‘Xiazhihong’ (XZH) and ‘Y2012-145’. SNPs are shown in grey or white background. (C) Part of protein sequence alignment of MrTPS20 in the four cultivars. (D) Protein 3D models for MrTPS20-BD and MrTPS20-XY predicted by SWISS-MODEL.

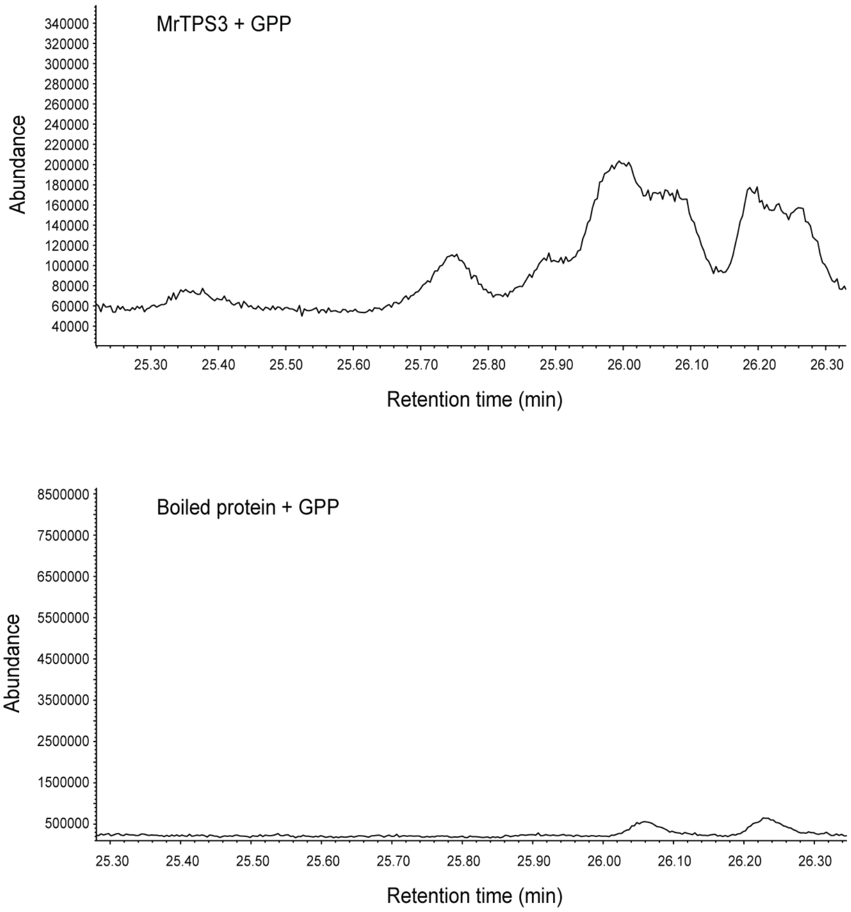

Fig. S11 Enzyme activity assay of MrTPS3 with substrate GPP. No α-pinene or β-caryophyllene was detected. Boiled protein was used as negative control. GC-MS was used for the detection.
