# Supplementary material for: MrTPS3 and MrTPS20 Are Responsible for β-Caryophyllene and α-Pinene Production, Respectively, in Red Bayberry (Morella rubra)
Source: Front Plant Sci. 2022 Jan 7;12:798086. doi: 10.3389/fpls.2021.798086 (PMC8777192; doi:10.3389/fpls.2021.798086)
Supplement: Supplementary file 2 [file Table_1.DOCX]

**Table S1. qPCR primers for *TPS* genes**

| Gene Name | Forward (5’ to 3’) | Reverse (5’ to 3’) |
| --- | --- | --- |
| *MrTPS1* | CGTCCTTTCTTGGAATGGGAGAAAC | TAACATTCGACAGCCGAGGCAATG |
| *MrTPS3* | GTTCCACCAGAAACACACACCTAC | CTCGAACGATTCTTCGGGAACATC |
| *MrTPS10* | CACGCAGCTATCTTTCGCAC | CTCTGGGAGCTGATCTACGC |
| *MrTPS11* | TACACTGTTGCTCTATGGTTTCGA | TCAAGATGAGTGGTAGTGAAAGCA |
| *MrTPS16* | AAGAATAAGCAAGCGAGGACTACT | CATTTCCTTTCAACCCCTCCAATC |
| *MrTPS20* | TGGCATACAAGCTCACAGTCAGAC | CATTGGTCACCAAACATTCCGACC |
| *MrPP2A* | TGTGGGAGGTGTTGAGCATG | GGGGCACTCGGATAAGCAAT |

**Table S2. Primers for plasmid construction**

| Primer Name | Sequence (5’ to 3’) |
| --- | --- |
| MrTPS3-GFP-F | AGCTCGGTACCCGGGATGTTGTCCCAGCATTCAGCAGAA |
| MrTPS3-GFP-R | CATGTCGACTCTAGATTGGGGCACAGGATTAACGAGTAA |
| MrTPS20-GFP-F | AGCTCGGTACCCGGGATGGATCTTCGCTGTCTTGC |
| MrTPS20-GFP-R | CATGTCGACTCTAGATCGAACATTGGTCACCAAACATTC |
| MrTPS3-pET32a-F | GCCATGGCTGATATCGGATCCATGTTGTCCCAGCATTCAGC |
| MrTPS3-pET32a-R | CTCGAGTGCGGCCGCAAGCTTTTATTGGGGCACAGGATTAACG |

| **Table S3. Reads information of RNA-seq data** | | | | |
| --- | --- | --- | --- | --- |
| Samples | Reads Number | Bases(bp) | GC(%) | Mapping Rate |
| BQS1-1 | 62156020 | 7769502500 | 48.95 | 75.80% |
| BQS1-2 | 51497100 | 6437137500 | 49.32 | 78.60% |
| BQS1-3 | 47929708 | 5991213500 | 48.98 | 79.30% |
| BQS2-1 | 59648678 | 7456084750 | 47.41 | 84.10% |
| BQS2-2 | 65555762 | 8194470250 | 47.26 | 83.50% |
| BQS2-3 | 61099244 | 7637405500 | 47.28 | 83.70% |
| BQS3-1 | 61520586 | 7690073250 | 49.61 | 85.50% |
| BQS3-2 | 52805150 | 6600643750 | 49.96 | 84.30% |
| BQS3-3 | 40509466 | 5063683250 | 50.14 | 81.90% |
| DKS1-1 | 53685064 | 6710633000 | 49.18 | 78.30% |
| DKS1-2 | 68059988 | 8507498500 | 48.30 | 81.10% |
| DKS1-3 | 47054808 | 5881851000 | 48.38 | 80.40% |
| DKS2-1 | 64810068 | 8101258500 | 47.16 | 83.50% |
| DKS2-2 | 64978528 | 8122316000 | 47.33 | 83.10% |
| DKS2-3 | 65337222 | 8167152750 | 48.05 | 81.90% |
| DKS3-1 | 51850128 | 6481266000 | 47.37 | 81.20% |
| DKS3-2 | 61811432 | 7726429000 | 47.52 | 83.80% |
| DKS3-3 | 62043514 | 7755439250 | 47.87 | 84.30% |
| XZHS1-1 | 67793818 | 8474227250 | 48.70 | 83.80% |
| XZHS1-2 | 67101528 | 8387691000 | 48.97 | 83.20% |
| XZHS1-3 | 65568570 | 8196071250 | 48.57 | 81.70% |
| XZHS2-1 | 65294548 | 8161818500 | 47.34 | 81.80% |
| XZHS2-2 | 48449612 | 6056201500 | 47.57 | 80.50% |
| XZHS2-3 | 66634564 | 8329320500 | 46.91 | 85.10% |
| XZHS3-1 | 46291796 | 5786474500 | 47.00 | 81.50% |
| XZHS3-2 | 55533848 | 6941731000 | 48.11 | 81.10% |
| XZHS3-3 | 66418512 | 8302314000 | 47.64 | 82.40% |
| Y2012-145S1-1 | 59875986 | 7484498250 | 47.67 | 84.20% |
| Y2012-145S1-2 | 59572620 | 7446577500 | 47.56 | 83.60% |
| Y2012-145S1-3 | 64040878 | 8005109750 | 47.58 | 84.30% |
| Y2012-145S2-1 | 69629432 | 8703679000 | 48.89 | 80.80% |
| Y2012-145S2-2 | 62208760 | 7776095000 | 47.58 | 82.90% |
| Y2012-145S2-3 | 51710716 | 6463839500 | 48.90 | 77.80% |
| Y2012-145S3-1 | 57467166 | 7183395750 | 47.69 | 85.60% |
| Y2012-145S3-2 | 65803856 | 8225482000 | 47.23 | 85.40% |
| Y2012-145S3-3 | 58834786 | 7354348250 | 47.55 | 85.10% |

| **Table S4. Number of DEGs between different samples** | | | |
| --- | --- | --- | --- |
| Group | Total | Up | Down |
| BQS1-VS-BQS2 | 5552 | 3995 | 1557 |
| BQS1-VS-BQS3 | 2079 | 1293 | 786 |
| BQS1-VS-DKS1 | 1769 | 1021 | 748 |
| BQS1-VS-XZHS1 | 1777 | 920 | 857 |
| BQS1-VS-Y2012-145S1 | 3878 | 1878 | 2000 |
| BQS2-VS-BQS3 | 4337 | 1812 | 2525 |
| BQS2-VS-DKS2 | 2158 | 1111 | 1047 |
| BQS2-VS-XZHS2 | 1373 | 732 | 641 |
| BQS2-VS-Y2012-145S2 | 2341 | 1307 | 1034 |
| BQS3-VS-DKS3 | 1284 | 1093 | 191 |
| BQS3-VS-XZHS3 | 3390 | 2079 | 1311 |
| BQS3-VS-Y2012-145S3 | 2912 | 2133 | 779 |
| DKS1-VS-DKS2 | 3528 | 2278 | 1250 |
| DKS1-VS-DKS3 | 3899 | 1937 | 1962 |
| DKS1-VS-XZHS1 | 818 | 340 | 478 |
| DKS1-VS-Y2012-145S1 | 3375 | 1344 | 2031 |
| DKS2-VS-DKS3 | 1702 | 924 | 778 |
| DKS2-VS-XZHS2 | 2043 | 975 | 1068 |
| DKS2-VS-Y2012-145S2 | 1787 | 1021 | 766 |
| DKS3-VS-XZHS3 | 3100 | 1440 | 1660 |
| DKS3-VS-Y2012-145S3 | 673 | 459 | 214 |
| XZHS1-VS-XZHS2 | 3842 | 2357 | 1485 |
| XZHS1-VS-XZHS3 | 2571 | 1709 | 862 |
| XZHS1-VS-Y2012-145S1 | 3963 | 1658 | 2305 |
| XZHS2-VS-XZHS3 | 2404 | 1628 | 776 |
| XZHS2-VS-Y2012-145S2 | 608 | 386 | 222 |
| XZHS3-VS-Y2012-145S3 | 393 | 269 | 124 |
| Y2012-145S1-VS-Y2012-145S2 | 1548 | 1086 | 462 |
| Y2012-145S1-VS-Y2012-145S3 | 1394 | 1107 | 287 |
| Y2012-145S2-VS-Y2012-145S3 | 1063 | 599 | 464 |

**Table S7. Information of *TPS* genes in red bayberry genome**

| Gene ID | Gene Name | Subfamily | Chr. No. | Start | End | Protein Length (aa) |
| --- | --- | --- | --- | --- | --- | --- |
| KAB1227457.1 | *MrTPS1* | TPS-a | Chr1 | 32709159 | 32711805 | 552 |
| KAB1221921.1 | *MrTPS2* | TPS-e/f | Chr2 | 7238273 | 7242939 | 807 |
| KAB1224296.1 | *MrTPS3* | TPS-a | Chr2 | 35640061 | 35642641 | 552 |
| KAB1215218.1 | *MrTPS4* | TPS-a | Chr4 | 1917457 | 1919712 | 477 |
| KAB1215220.1 | *MrTPS5* | TPS-a | Chr4 | 1961894 | 1964278 | 512 |
| KAB1215225.1 | *MrTPS6* | TPS-a | Chr4 | 2043492 | 2045894 | 545 |
| KAB1215300.1 | *MrTPS7* | TPS-a | Chr4 | 2873720 | 2876084 | 426 |
| KAB1215301.1 | *MrTPS8* | TPS-a | Chr4 | 2879032 | 2881388 | 249 |
| KAB1215316.1 | *MrTPS9* | TPS-a | Chr4 | 2971583 | 2975652 | 509 |
| KAB1216361.1 | *MrTPS10* | TPS-a | Chr4 | 16895505 | 16898423 | 560 |
| KAB1216628.1 | *MrTPS11* | TPS-a | Chr4 | 20123817 | 20127220 | 567 |
| KAB1216859.1 | *MrTPS12* | TPS-e/f | Chr4 | 22586216 | 22590678 | 785 |
| KAB1210104.1 | *MrTPS13* | TPS-b | Chr6 | 17456904 | 17459733 | 568 |
| KAB1210106.1 | *MrTPS14* | TPS-b | Chr6 | 17487947 | 17490495 | 508 |
| KAB1210108.1 | *MrTPS15* | TPS-b | Chr6 | 17564400 | 17566877 | 567 |
| KAB1210117.1 | *MrTPS16* | TPS-b | Chr6 | 17696178 | 17698596 | 538 |
| KAB1210118.1 | *MrTPS17* | TPS-b | Chr6 | 17708971 | 17720028 | 439 |
| KAB1210636.1 | *MrTPS18* | TPS-c | Chr6 | 22261343 | 22268463 | 808 |
| KAB1205510.1 | *MrTPS19* | TPS-b | Chr7 | 5855344 | 5857855 | 603 |
| KAB1205519.1 | *MrTPS20* | TPS-b | Chr7 | 5963735 | 5965594 | 620 |
| KAB1205521.1 | *MrTPS21* | TPS-b | Chr7 | 5975550 | 5976776 | 254 |
| KAB1205568.1 | *MrTPS22* | TPS-b | Chr7 | 6453029 | 6456646 | 473 |
| KAB1205569.1 | *MrTPS23* | TPS-b | Chr7 | 6486778 | 6489170 | 481 |
| KAB1206135.1 | *MrTPS24* | TPS-b | Chr7 | 14224414 | 14226645 | 410 |
| KAB1206529.1 | *MrTPS25* | TPS-b | Chr7 | 20496560 | 20498791 | 395 |
| KAB1202075.1 | *MrTPS26* | TPS-e/f | Chr8 | 3387407 | 3392176 | 838 |
| KAB1202340.1 | *MrTPS27* | TPS-a | Chr8 | 6072219 | 6074305 | 294 |
| KAB1202345.1 | *MrTPS28* | TPS-a | Chr8 | 6120693 | 6122272 | 218 |
| KAB1203283.1 | *MrTPS29* | TPS-c | Chr8 | 17843424 | 17850405 | 799 |
| KAB1201248.1 | *MrTPS30* | TPS-b | Scaffold_16 | 156680 | 159174 | 568 |
| KAB1201252.1 | *MrTPS31* | TPS-g | Scaffold_16 | 194362 | 198093 | 540 |
| KAB1201253.1 | *MrTPS32* | TPS-g | Scaffold_16 | 208563 | 213338 | 461 |
| KAB1201254.1 | *MrTPS33* | TPS-g | Scaffold_16 | 219271 | 222958 | 540 |
| KAB1201255.1 | *MrTPS34* | TPS-g | Scaffold_16 | 232264 | 237493 | 719 |
